# Structure Characterization and Dye Adsorption Properties of Modified Fiber from Wheat Bran

**DOI:** 10.3390/molecules29112581

**Published:** 2024-05-30

**Authors:** Wenbin Quan, Juan Wang, Jihong Huang, Dale Zhang

**Affiliations:** 1 Food and Pharmacy College, Xuchang University, Xuchang 461000, China; 2 School of Food Science and Engineering, South China University of Technology, Guangzhou 510641, China; 3 Collaborative Innovation Center of Functional Food by Green Manufacturing, Xuchang 461000, China; 4 College of Agriculture, Henan University, Kaifeng 475001, China

**Keywords:** wheat bran fibers, chemical modification, dye adsorption, adsorption kinetics, thermodynamics analysis

## Abstract

The fibers from four wheat varieties (FT, XW 26, XW 45, and KW 1701) were selected and chemically modified with NaOH, epichlorohydrin, and dimethylamine to improve the adsorption capacity for anionic dye. The structure of the fibers with or without modification was characterized by scanning electron microscope (SEM), X-ray diffraction (XRD), and Fourier-transform infrared (FTIR) spectrometry. The modified products were studied from the aspects of adsorption capacities, adsorption kinetics, and thermodynamics to provide a reference for the utilization of wheat bran. By SEM, more porous and irregular structures were found on the modified fibers. The XRD results showed that the crystals from the original fibers were destroyed in the modification process. The changes in fibers’ infrared spectra before and after modification suggested that quaternary ammonium salts were probably formed in the modification process. The maximum adsorption capacity of wheat bran fibers for Congo red within 120 min was 20 mg/g for the unmodified fiber (XW 26) and 93.46 mg/g for the modified one (XW 45). The adsorption kinetics of Congo red by modified wheat bran fiber was in accord with the pseudo-second-order kinetic model at 40 °C, 50 °C, and 60 °C, indicating that the adsorption process might be mainly dominated by chemisorption. The adsorption was more consistent with the Langmuir isothermal adsorption model, implying that this process was monolayer adsorption. The thermodynamic parameters suggested that the adsorption occurred spontaneously, and the temperature increase was favorable to the adsorption. As mentioned above, this study proved that the wheat bran fiber could possess good adsorption capacities for anion dye after chemical modification.

## 1. Introduction

Wheat is one of the most widely planted grains in the world, with an annual output of more than 650 million tons [1], becoming a significant source of calories for humans [2]. Wheat bran is the outer part of wheat grains after milling [3], accounting for about 25% of the original grain weight [4]. However, wheat bran is currently mainly used in fields such as feed, fermentation, etc. The utilization of wheat bran is insufficient, and its application value needs to be further explored. Therefore, developing the deep utilization of wheat bran can effectively reduce waste and generate economic value for the wheat industry.

With the development of global industrialization, water pollution is becoming more and more serious; among which, dye is one of the most widespread water pollutants [5]. It is estimated that up to 1 × 10^6^ tons of dye is produced globally each year [6], and about 12% of synthetic dye is lost during manufacturing and processing, 20% of which enters the environment with industrial wastewater [7]. The textile industry alone can emit 146,000 tons of dyes and their industrial effluents each year [8]. The treatment of dye wastewater is a challenging problem due to its massive amount and stable toxic pollutants [9]. Some dyes, such as methylene blue and basic fuchsin, have toxic effects, such as carcinogenicity and hypertension [10], so the treatment of dye wastewater deserves attention. Congo red is a typical anionic azo dye with carcinogenic and mutagenic effects widely utilized in textile industries [11,12]. Therefore, the presence of Congo red in dye wastewater should not be ignored.

Although some advanced dye removal technologies such as electrochemical oxidation [13] and filtration [14] have been invented, their application in industry has been limited due to high cost and other factors. Currently, the adsorption technique is one of the most widely used methods of sewage disposal, but the commonly used adsorbents such as activated carbon are relatively expensive and difficult to regenerate [15]. Recently, agricultural by-products have become an important material for developing alternative dye adsorbents owing to their massive amounts, low cost, and large number of pores. Many agricultural wastes after chemical modification have shown potential as dye adsorbents, and the dye adsorption capacity of wheat bran has been proven [16]. But the capacities of many other varieties of wheat bran are still unclear. Although natural agricultural by-products show a certain dye adsorption capacity, they often need to be improved by chemical or physical modification to be used as dye adsorbents. Through chemical modification, active groups can be introduced into agricultural by-products, changing the crystal structure of raw material cellulose and generating more loose and porous structures, which is helpful to improve the dye adsorption performance of adsorbents and broaden the application of agricultural by-products. At present, there are few studies on the chemical modification of the fiber in wheat bran, and research on its use for dye adsorption is even rarer.

In this study, wheat bran fiber was chemically modified with NaOH, epichlorohydrin, and dimethylamine, and the structure and dye adsorption performance before and after modification were compared, proving their potential to be dye adsorbents. The adsorption kinetics and thermodynamic properties of fiber were also studied. Considering that the research on the application of wheat bran to dye adsorption is very rare at present, it is believed that the findings of this study can accumulate data on different varieties of wheat bran, supplement the understanding of wheat bran fiber, and provide the theoretical reference for the application of wheat bran in the field of dye adsorption, which is conducive to increasing the added value of wheat bran, and reduce the waste of wheat bran.

## 2. Results and Discussion

### 2.1. Basic Compositions and Total Dietary Fiber Contents of Wheat Bran

The basic compositions of the four varieties of wheat bran are shown in Table 1. The total carbohydrate, protein, and ash contents of XW 26, XW 45, and KW 1701 bran were similar. In contrast, the protein (19.5%) and ash (6.5%) contents of FT wheat bran were much higher than those of the other three varieties, while the total carbohydrate (58.8 g/100 g) was slightly lower. In addition, the fat content of XW 26 (1.5 g/100 g) was slightly lower than that of other varieties, and the total energy contents of the four kinds of wheat bran exceeded 1400 KJ/100 g. Generally speaking, the outer parts of the grains usually contained more dietary fiber, while the endosperm had a lower dietary fiber content [17]; therefore, wheat bran may have the ability to be one of the sources to obtain dietary fiber. The contents of TDF (58.49 g/100 g) in FT wheat bran were considerably higher than those in the other three varieties, while XW 45 bran had the least amount of TDF (27.77 g/100 g). To sum up, the dietary fiber contents of wheat bran from different sources varied, but the total dietary fiber contents of the four kinds of wheat bran were more than 25 g/100 g, indicating that they possessed a certain potential to be dietary fiber sources. Considering that there are wrinkles and pores on the surface of the fiber [18], wheat bran fiber might have a stronger dye adsorption capacity than the original wheat bran and could be used as dye adsorbent. The differences in the basic compositions of the four kinds of wheat bran might affect fiber contents and properties, causing diversity in the dye adsorption capacity.

### 2.2. Monosaccharide Composition Analysis

The structure and active function of fibers may be affected by the composition of the monosaccharides, and therefore, the determination of the monosaccharide composition can improve the understanding of wheat bran fibers. Table 2 presents the molar ratios of the monosaccharide compositions of the four wheat bran fibers, suggesting that there were a few differences in the types and contents of the monosaccharides of different varieties of wheat bran fibers. Arabinose, galactose, glucose, and xylose were detected in all four wheat bran fibers. Except that the xylose content of FT wheat bran fiber (47.60%) was higher than that of glucose (25.01%), the order of the monosaccharide contents of the other three wheat bran fibers was glucose > xylose > arabinose > galactose. Considering that the main components of hemicellulose include xylose, arabinose, glucose, galactose, etc., it can be inferred that all four wheat bran fibers contained a certain amount of hemicellulose. It was noted that a low level of rhamnose was detected in the FT fiber, which may be associated with pectin components. The difference in monosaccharide compositions of the four varieties of bran fibers suggested that the contents of the components differed, which, in turn, might have had an impact on their properties and dye adsorption capacities.

### 2.3. Structure Characterization

#### 2.3.1. Optical Microscope and Scanning Electron Microscope Observation

**Figure 1:**
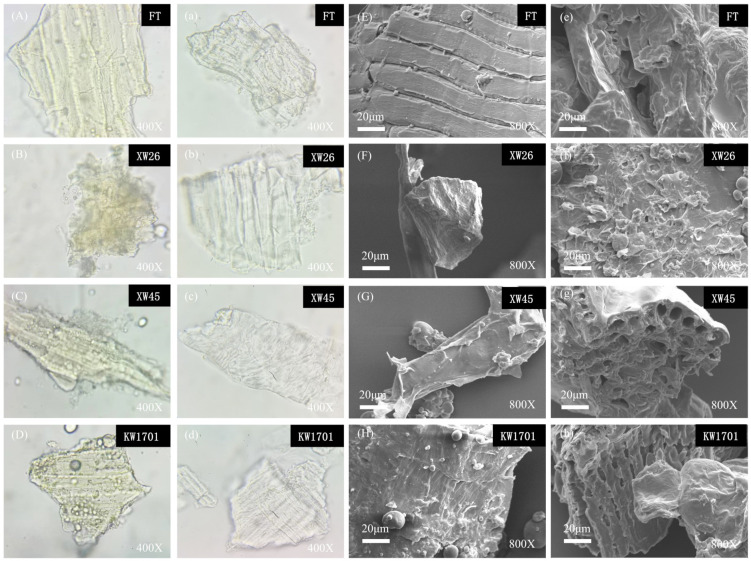
The morphology of wheat bran fibers before (**A**–**H**) and after modification (**a**–**h**).

SEM is a commonly used technique to observe the morphology and microstructure of sample surfaces. Unlike optical microscopy, SEM uses high-energy electron beams instead of light to illuminate the sample, resulting in higher resolution [19]. The fibers from four kinds of wheat bran before and after modification were observed by optical microscope and scanning electron microscope at 400× and 800× magnification, respectively, and the results are shown in Figure 1. Figure 1A–H present the microstructures of the wheat bran fibers. The original fibers were composed of lamellae with folds on the surface. In comparison, as shown in Figure 1a–h, the modified wheat bran fibers were noticeably lighter in color and had looser, more irregular, and porous structures. It could expose more quaternary ammonium salts to anionic dye molecules, which was conducive to dye adsorption. The loose and porous structures might be due to the alkali treatment, which destroyed the hydrogen bond, causing the swelling of cellulose, the increase in surface area, and the removal of lignin [20]. The alkali hydrolysis, etherification, and amination reactions during the modification process may lead to swollen and irregular structures on the surface of wheat bran fibers. In summary, the structures of the fibers from the four varieties of wheat bran changed significantly before and after modification, and the modified ones possessed more cavities and became more irregular, which might be attributed to the chemical modification on the fibers. Such looser and more porous microstructures might contribute to a better adsorption capacity for dyes. It should be noted that, although the surface and microstructure of the fibers can be observed by SEM, BET analysis was not included in this study to provide detailed information about the pore structure and specific surface area of the samples. Therefore, in the future, BET analysis can be performed to gain further insight into the microscopic properties of the fibers. This will facilitate a more comprehensive assessment of the effects of the modification process on the surface area and pore structure of the wheat bran fibers.

#### 2.3.2. Fourier-Transform Infrared Spectroscopy Analysis

**Figure 2:**
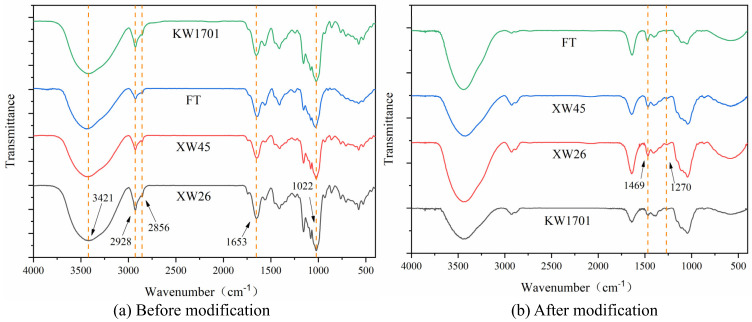
Infrared spectra of wheat bran fibers before and after modification.

Fourier-transform infrared spectroscopy is a frequently used approach to identify the functional groups and chemical bonds of compounds according to the spectra [21]. The FTIR spectra of the fibers from the four kinds of wheat bran before and after modification are depicted in Figure 2, and the wavenumbers of most of their absorption bands were quite similar. According to the literature [22], broad and strong absorption bands of the fibers of the four wheat bran appeared near 3421 cm^−1^, which were attributed to O-H stretching vibration. Referring to the study [23], the weak signals at 2928 cm^−1^ and 2856 cm^−1^ might originate from the C-H stretching vibration of methylene, and the absorptions near 1410 cm^−1^ were ascribed to the bending vibration of C-H. With reference to the research [24,25], the bands near 1653 cm^−1^ were assigned to C=O stretching vibration, indicating that there was uronic acid or an ester group (-COOR). As reported previously [25], The strong signals near 1022 cm^−1^ corresponded to C-O stretching vibration, which might be derived from the C-O-H and C-O-C structures of the sugar ring. The presence of the above functional groups indicated that wheat bran fibers had the typical structure of polysaccharide compounds [24]. The infrared spectra of wheat bran fibers were similar to that of fibers from other sources, such as Japanese grape pomace, oats, enoki mushrooms, etc. [26,27]. Their rich oxygen-containing functional groups can facilitate dye adsorption through electrostatic interactions and hydrogen bonds [28]. According to Figure 2a, there were hydroxyl groups in wheat bran fibers, which could bring in functional groups by chemical reaction, resulting in the good modification potential of wheat bran fibers. Based on the principle of the modification technique, a C-N bond and N-H bond might be formed during this process. Considering that the absorption peak of N-H stretching vibration may occur in the range of 3500 cm^−1^~3300 cm^−1^ [29], the bending vibration of N-H may occur in the range of 1650 cm^−1^~1550 cm^−1^ [29], and the absorption peak strength of the modified product was enhanced in these wavenumber ranges, they might be the results of the influence of N-H introduced by an amination reaction on the absorption peaks. After modification, new absorption peaks appeared at 1469 cm^−1^ and 1270 cm^−1^, as reported in References [29,30,31], which may be attributed to the stretching vibration of C-N. In summary, the changes in the fibers’ infrared spectra before and after modification suggested that quaternary ammonium salts were probably formed in the modification process, which might enhance the adsorption capacity for anionic dyes.

#### 2.3.3. X-ray Diffraction Analysis

**Figure 3:**
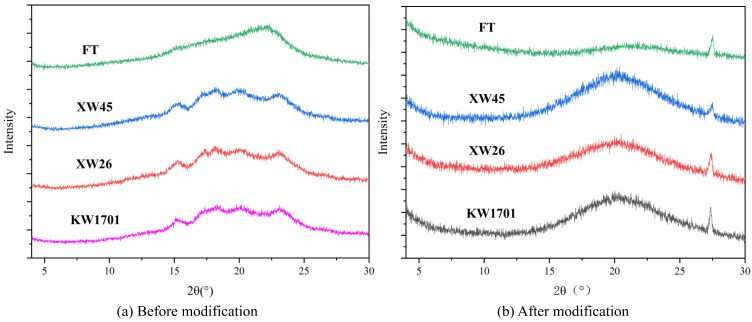
X-ray diffraction patterns of wheat bran fiber before and after modification.

X-ray diffraction is an important means to analyze the crystallization of samples [32]. The X-ray diffraction patterns of four kinds of wheat bran fibers before and after modification are presented in Figure 3. Relatively strong and sharp diffraction peaks near 15° and 23° are observed in Figure 3a, which are considered characteristic peaks of cellulose type I [crystal face 10$\bar{1}$ and 002] [33]. It can be concluded that the fibers of wheat bran from the four sources were mainly composed of type I cellulose crystals, but there were differences in the crystallinity and the distribution of crystalline and non-crystalline regions. As for Figure 3b, the modified wheat bran fibers have no diffraction absorption peak at the relevant angles, and there are only wide peaks at about 20°, which might come from the amorphous part [22,34]. This result was similar to the pattern of bamboo treated with epichlorohydrin [35], indicating that chemical reactions destroyed the original crystals of wheat bran fiber. In addition, the XRD patterns of modified wheat bran fibers showed a diffraction peak near 27° and the jackfruit peel residue cellulose with a similar modification [36] also possessed a diffraction peak at the same angle. To sum up, the crystals of wheat bran fibers were destroyed by the modified reactions, and the modified products were mainly composed of amorphous structures.

### 2.4. Dye Adsorption Capacities of Wheat Bran Fibers before and after Modification

**Figure 4:**
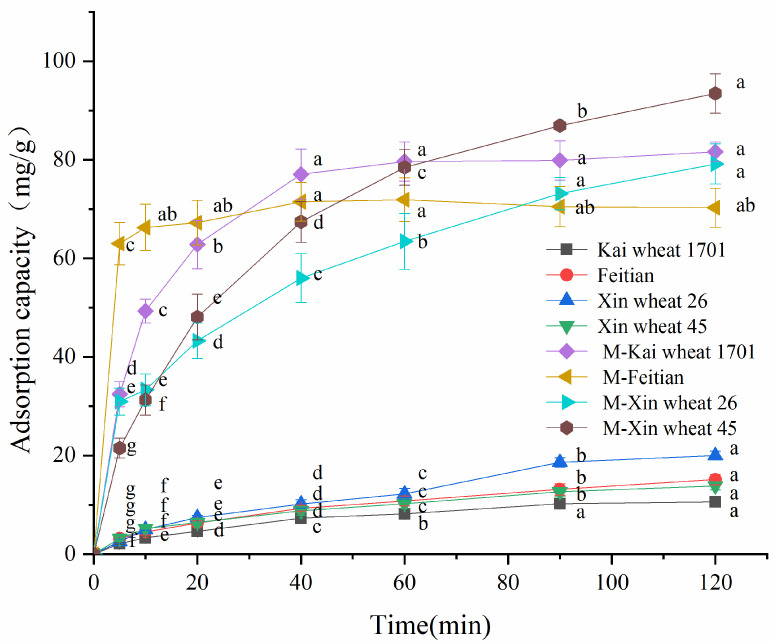
The adsorption capacity of wheat bran fibers to Congo red before and after modification (where fibers with “M-” were the modified ones). Different letters indicate that the adsorption capacities of the same wheat bran fiber at different times were significantly different (*p* < 0.05).

The adsorption capacities of wheat bran fibers before and after the modification to Congo red are compared in Figure 4. It can be seen that the adsorption capacities of modified wheat bran fibers for Congo red were significantly improved compared to those before modification. The maximum capacity of unmodified wheat bran fiber within 120 min was only 20 mg/g, which was slightly stronger than the capacity of rice husk [37] for Congo red and close to that of cattail root [38], while the adsorption capacity of modified wheat bran fiber could exceed 90 mg/g within 120 min. This considerable increase in capacity might be the result of the fact that the adsorption of the unmodified fibers was mainly based on physical adsorption, while cationic-modified products could facilitate the adsorption of anionic dyes. Moreover, it could be observed that, compared to FT, the adsorption capacities of XW 45, KW 1701, and XW 26 fibers were improved more after modification. Combined with the data of the fiber monosaccharide composition, it was found that the glucose contents of these three kinds of fibers were higher. It is well known that glucose is hexose, while xylose and arabinose, which are also rich in wheat bran fibers, are pentose. Therefore, glucose contains more active hydroxyl groups. Considering that epichlorohydrin will react with the hydroxyl group during the modification process, the higher glucose content might be more conducive to the modification reaction. Differences in the composition of monosaccharides might be the reason for the diversities in the enhancement of the adsorption capacity. To sum up, amination modification can effectively improve the adsorption performance of wheat bran fibers to anionic dyes.

### 2.5. Adsorption Kinetics

**Figure 5:**
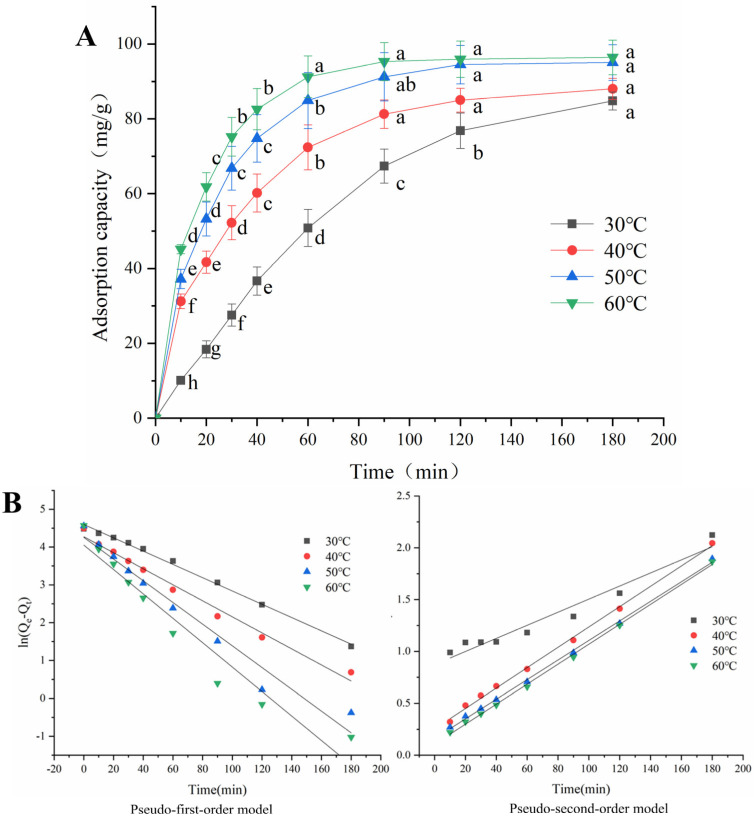
(**A**) Adsorption kinetics of the modified XW 45 fiber. Different letters indicate that the adsorption capacities of the modified fiber at the same temperature at different times were significantly different (*p* < 0.05). (**B**) Pseudo-first-order model and pseudo-second-order model of Congo red adsorption by the modified XW 45 fiber.

According to Figure 4, the modified XW 45 had the strongest adsorption capacity at 120 min, so it was selected as the research object in the following parts, and its adsorption kinetics of Congo red at different temperatures are presented in Figure 5A and Table 3. According to Figure 5A, the adsorption capacity of modified fiber for Congo red increased rapidly at the initial stage. Then, the growth slowed down and the capacity finally became constant. The adsorption kinetics curves at different temperatures were compared, and it was found that the rising temperature was beneficial to the adsorption of modified fiber for Congo red to a certain extent. At 30 °C, the adsorption capacity increased slowly, and it only reached 36.65 mg/g within 40 min. When the temperature rose, the adsorption capacity increased significantly. As depicted in Figure 5A, at 40 °C, 50 °C, and 60 °C, the higher the temperature was, the faster the adsorption capacity ascended. At 60 °C, the adsorption capacity surged to 82.59 mg/g at 40 min and approached the maximum (> 95 mg/g) at 90 min. This might be due to the increase in temperature, which promoted chemisorption between Congo red and the modified fiber with cationic groups.

According to Table 3, at 30 °C, although the R^2^ values of the two models both exceeded 0.95, the R^2^ of the pseudo-first-order kinetic model was higher, and the calculated $Q_{e}$ of the pseudo-first-order kinetic model was closer to the experimental one, which suggested that the adsorption process followed to the pseudo-first-order kinetic model. At 40 °C, 50 °C, and 60 °C, the R^2^ values of the pseudo-second-order kinetic model were more than 0.99, indicating that the adsorption of modified wheat bran fiber on Congo red was more consistent with the pseudo-second-order kinetic model. Many studies on the dye adsorption of chemically modified agricultural waste [39] also found that the dye adsorption processes were better suited to the pseudo-second-order kinetic model than the pseudo-first-order kinetic model. As for the calculated $Q_{e}$, at 40 °C, 50 °C, and 60 °C, the calculated $Q_{e}$ values of the pseudo-second-order kinetic model were closer to the experimental results, while the calculated $Q_{e}$ values fitted by the pseudo-first-order kinetic model were quite different from the experimental values. The adsorption rate constant $k_{2}$ of the pseudo-second-order kinetic model increased with the temperature. The above results indicated that the adsorption of the modified wheat bran fiber on Congo red might be a predominantly chemical adsorption process. At 30 °C, the chemicals reacted slowly owing to the low temperature, and chemisorption was not strong enough to dominate the process; thus, the adsorption rate of the modified fiber on Congo red was low, and the adsorption was still in the initial stage during the determination time, which was more consistent with the pseudo-first-order kinetic model. When the temperature was over 40 °C, the positive correlation between $k_{2}$ and temperature implied that the temperature rise accelerated the chemical reaction, and chemical adsorption played a significant role, which followed the pseudo-second-order kinetic model. It was also proven that the amination reaction occurred on the modified wheat bran fiber, thus improving its adsorption capacity for Congo red.

### 2.6. Adsorption Isotherms

**Figure 6:**
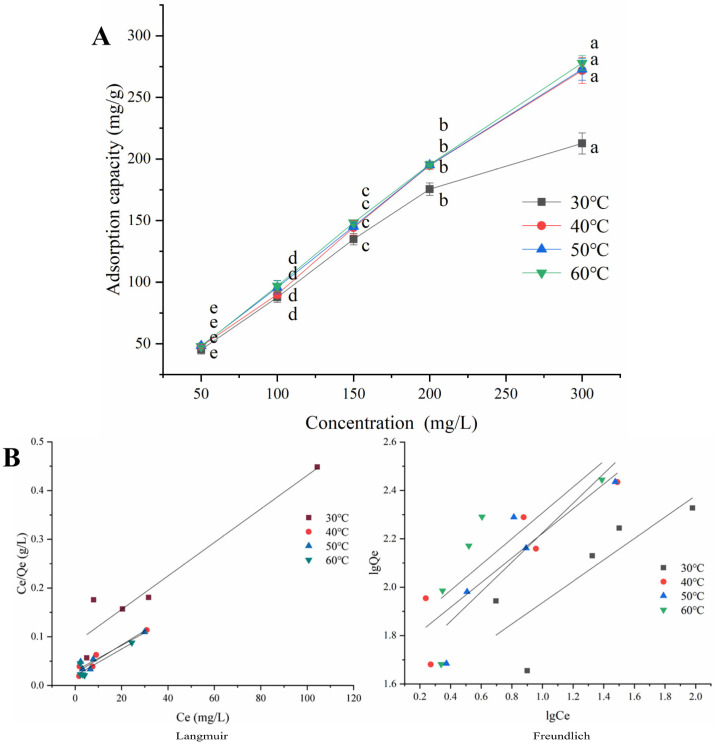
(**A**) Adsorption isotherms of the modified XW 45 fiber for Congo red. Different letters indicate that the adsorption capacity of the modified fiber with different initial concentrations at the same temperature had significant differences (*p* < 0.05). (**B**) Langmuir and Freundlich isothermal adsorption model fitting for Congo red adsorption by the modified XW 45 fiber.

Modified XW 45 bran fiber was used as the research object, and the isothermal adsorption lines at 30 °C, 40 °C, 50 °C, and 60 °C are plotted in Figure 6. In general, the adsorption capacity of the modified XW 45 bran fiber for dye at equilibrium increased with the rise of the initial concentration of the solution, and the higher the temperature was, the higher the equilibrium adsorption capacity of the modified product at the same initial concentration. At 30 °C, the equilibrium adsorption capacities of the modified adsorbent at different concentrations were the lowest. Under the concentration of 300 mg/L, the equilibrium adsorption capacity was only about 212 mg/g, while, at 60 °C, it was significantly higher, and there was an approximately linear positive correlation between the capacity and the initial concentration of the solution (R^2^ = 0.9974). The adsorption capacity in 300 mg/L dye solution at 60 °C surged to 278 mg/g. In summary, the modified wheat bran fiber had a good adsorption capacity for Congo red in the range of 50~300 mg/L, and the adsorption capacity could be improved by moderate heating.

According to the parameters in Table 3, the R^2^ values of the Langmuir isothermal model were higher than that of the Freundlich isothermal model at 30 °C, 40 °C, 50 °C, and 60 °C. The R^2^ of the Langmuir model exceeded 0.9 at 30 °C, 40 °C, and 50 °C, indicating that the Langmuir isothermal model was used to describe the adsorption behavior of the modified XW 45 fiber on Congo red precisely. This result was similar to the findings of chemically modified dye adsorbents derived from orange peel and orange tree sawdust [40,41]. At the temperatures of 40 °C and above, the maximum monolayer adsorption capacity $Q_{m}$ values of the Langmuir isothermal model were over 300 mg/g, suggesting that the modified wheat bran fiber had a strong adsorption capacity for Congo red dye at a high initial concentration. To sum up, the modified wheat bran fiber had an impressive adsorption capacity for Congo red, and the experimental data were better fitted to the Langmuir isothermal model than the Freundlich model in the concentration range of 50 to 300 mg/L.

### 2.7. Adsorption Thermodynamic Parameters

The calculations were based on data from the adsorption isotherms study with an initial dye concentration of 300 mg/L, and the parameters are listed in Table 4. At different investigated temperatures, the negative Gibbs free energy change ∆G° implied that the adsorption of Congo red by the modified wheat bran fiber was a spontaneous process. The enthalpy change ∆H° was 55.10 KJ·mol^−1^, and it suggested the endothermic nature of the adsorption process, which meant the rise in temperature could enhance the adsorption. This conclusion was in good agreement with the phenomenon observed in the experiment. The enthalpy change ∆H ranged from 20.19 to 418.14 KJ·mol^−1^, indicating that the adsorption process might be chemisorption [42]. The entropy change ∆S° was 198.61 J·mol^−1^·K^−1^, denoting that the chaos increased after the dye was adsorbed on the modified wheat bran fiber, which might be due to the chemical interaction between the dye and the modified adsorbent. In summary, although we have studied the adsorption process of Congo red by modified wheat bran fibers in terms of adsorption kinetics and adsorption thermodynamics, it should be noted that it is difficult to propose a relatively complete adsorption mechanism based on the current research in this paper. This is because we lack the characterization of the modified wheat bran fibers after dye adsorption. Some characterizations can provide information about the adsorbent surface structural changes and the chemical reactions that may occur during adsorption, which are important for understanding the adsorption mechanism and optimizing the adsorption process.

## 3. Materials and Methods

### 3.1. Materials and Reagents

Feitian (FT) wheat bran was obtained from Henan Feitian Biotechnology Co., Ltd. (Hebi, China); Xin wheat 26 (XW 26) wheat bran, Xin wheat 45 (XW 45) wheat bran, and Kai wheat 1701 (KW 1701) wheat bran were supplied by the College of Agriculture, Henan University (Kaifeng, China). The total dietary fiber assay kit was purchased from Megazyme Co., Ltd. (Bray, Ireland). 2-Morpholinoethanesulphonic acid (MES) was purchased from Shanghai Yuanju Bio-tech Co., Ltd. (Shanghai, China). Tris (hydroxymethyl) aminomethane was purchased from Sinopharm Chemical Reagent Co., Ltd. (Shanghai, China). Potassium bromide (KBr) was purchased from Tianjin Kemiou Chemical Reagent Co., Ltd. (Tianjin, China). Amylase, protease, and amyloglucosidase were purchased from Novozymes Co., Ltd. (Copenhagen, Denmark). Congo red and sodium hydroxide (NaOH) were purchased from Fuchen Chemical Reagent Co., Ltd. (Tianjin, China). Epichlorohydrin was purchased from Shanghai Lingfeng Chemical Reagent Co., Ltd. (Shanghai, China). Dimethylamine solution (33%) was purchased from Shanghai Chemical Reagent Purchase Supply Wulian Chemical Plant Co., Ltd. (Shanghai, China). Methanol and trifluoroacetic acid were purchased from Anpel Laboratory Technologies Inc. (Shanghai, China). The other chemicals and reagents were purchased from Guangdong Guangshi Regent Technology Co., Ltd. (Zhaoqing, China). All commercial chemicals and reagents used were of analytical grade unless mentioned.

### 3.2. The Basic Composition of Wheat Bran

The moisture, ash, protein, and fat contents of wheat bran were determined according to the methods from the Chinese national standards, including GB 5009.3-2016 [43], GB 5009.4-2016 [44], GB 5009.5-2016 [45], and GB 5009.6-2016 [46]. The total carbohydrate and energy contents were calculated using the following formulae:

(1) Total Carbohydrate=100−Water−Ash−Protein−Fat

(2) Energy=Protein×17+Fat×37+Carbohydrate×17

### 3.3. Determination of Dietary Fiber Content

The Megazyme dietary fiber assay kit (Megazyme Co., Ltd., Bray, Ireland) was used to determine the contents of the total dietary fiber (TDF) according to the method AOAC 996.43. In this process, the protein contents of the residues were analyzed by the Kjeldahl method on a Kjeltec Auto Analyzer (Shandong Hanon Scientific Instruments Co., Jinan, China) using N × 6.25 as the conversion factor. As for the ash analysis of the residue, the sample was incinerated in a high-temperature furnace (Yamato Scientific Co., Tokyo, Japan) for 5 h at 525 °C. The contents of the dietary fiber were calculated by the following equations:

(3) $$m_{R}=m_{GR}-m_{G}$$

(4) $$m_{B}=\bar{m_{BR}}-m_{BP}-m_{BA}$$

(5) $$X=\frac{\bar{m_{R}}-m_{P}-m_{A}-m_{B}}{\bar{m}}\times 100$$

where $m_{R}$ = residue weights (g) for samples; $m_{GR}$ = weights (g) for crucibles, celite, and residues; $m_{G}$= weights (g) for crucibles and celite; $m_{B}$ = residue weights (g) for blank determinations; $\bar{m_{BR}}$ = average residue weights (g) for duplicate blank determinations; $m_{BP}$ and $m_{BA}$ = weights (g) of protein and ash, respectively, determined on the two blank residues; X = contents (g/100 g) of TDF; $\bar{m_{R}}$ = average residue weights (g) for duplicate samples; $m_{P}$ and $m_{A}$ = weights (g) of protein and ash, respectively, determined on the two residues; $\bar{m}$ = weights (g) for the samples.

### 3.4. Preparation of Wheat Bran Fiber

The extraction method of fiber from wheat bran was slightly changed from the published procedure [47], and the main steps were as follows.

The milled wheat bran was soaked in water at 45 °C to remove the sugar before being centrifuged at 3000 r/min for 10 min. Then, the supernatant was discarded, and distilled water was added to the precipitate. Amylase was added to the mixture and incubated in a constant temperature water bath oscillator (Changzhou Aohua Instrument Co., Ltd., Changzhou, China) at 50 °C for 2 h. The amount of amylase was 0.2%, and the pH of the mixture was adjusted to 4.0. After the incubation, glucoamylase was added. The amount of glucoamylase was 0.2%, and the pH was adjusted to 4.0 as well. The mixture was incubated at 50 °C for 2 h again. Subsequently, it was centrifuged at 3000 r/min for 10 min, and the supernatant was removed. Then, distilled water was added with the protease to the precipitate. The amount of protease was 0.2%, but the pH was adjusted to 7.0. Then, the blend was incubated in the water bath at 50 °C for 2 h. After the reaction, the blend was centrifuged at 3000 r/min for 10 min, and the precipitate was freeze-dried for 40 h, crushed, and sieved through 80 mesh to obtain the fiber sample.

### 3.5. Monosaccharide Composition Analysis

For the analysis, 1 mL of trifluoroacetic acid (2M) was added to 3 mg of fiber sample, and the mixture was incubated at 121 °C for 2 h. Then, the sample was dried with nitrogen. After methanol was added to the residue, it was blown dry again and repeated 2–3 times. Afterward, the residue was dissolved in water and filtered through a 0.22 μm microporous filtering film for determination by a high-performance anion-exchange chromatography (HPAEC) system (Thermo Fisher Scientific Inc., MA, USA) using a pulsed amperometric detector [48].

HPAEC conditions: Dionex™ CarboPac™ PA20 (150*3.0 mm, 10 μm) liquid chromatography column; injection volume, 5 μL; solvent system A (H_2_O), solvent system B (0.1 M NaOH), and solvent system C (0.1 M NaOH and 0.2 M NaAc); flow rate, 0.5 mL/min; column temperature, 30 °C; gradient program: at 0 min, A/B/C (95:5:0, *v*/*v*/*v*); at 26 min, A/B/C (85:5:10, *v*/*v*/*v*); at 42 min, A/B/C (85:5:10, *v*/*v*/*v*); at 42.1 min, A/B/C (60:0:40, *v*/*v*/*v*); at 52 min, A/B/C (60:40:0, *v*/*v*/*v*); at 52.1 min, A/B/C (95:5:0, *v*/*v*/*v*); at 60 min, A/B/C (95:5:0, *v*/*v*/*v*).

### 3.6. Modification Techniques of Fiber

The main steps revised from the published method [42,49] were as follows.

**Figure 7:**
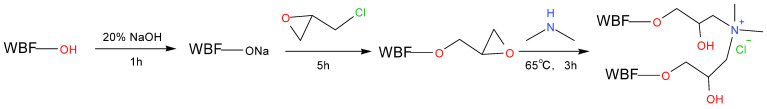
The schematic diagram of the modification process (WBF represents the backbone of the compounds in wheat bran fibers).

The appropriate amount of wheat bran fiber was alkalized by 20% NaOH solution at a ratio of material to liquid (*m*/*v*) of 1:10 for 1 h before being centrifugated at 3000 r/min for 10 min and filtered. Then, epichlorohydrin was added at the ratio of material to liquid (*m*/*v*) 1:5 and magnetically stirred at room temperature for 5 h. After that, filtration was performed, and the residue was washed with water and acetone. Subsequently, dimethylamine solution was added under the ratio of material to liquid (*m*/*v*) of 1:6, and the blend was stirred at 65 °C for 3 h. Finally, it was filtered, and the residue was washed with an appropriate amount of water and acetone and dried to obtain the modified product. The schematic diagram of the modification process is shown in Figure 7.

### 3.7. Structure Characterization of Wheat Bran Fibers

#### 3.7.1. Optical Microscopic Observation

A small amount of wheat bran fiber samples was taken and dispersed in ethanol. Then, 2~3 drops of the mixture were put on a slide, and the morphologies of wheat bran fiber were observed by a biological optical microscope (Shanghai Yoke Instrument Co., Ltd., Shanghai, China) after covering the slide.

#### 3.7.2. Scanning Electron Microscopic Observation

With reference to the published method [50], a small amount of sample powder was evenly distributed and coated with a layer of gold on the conductive tape affixed to a stage. Then, the stage was placed, vacuumed, observed, and photographed under 10 KV acceleration voltage and 800× magnification in the scanning electron microscope (Zeiss Co., Ltd., Oberkochen, Germany).

#### 3.7.3. X-ray Diffraction

According to the reported approach [50], a X-ray diffractometer (Malvern Panalytical B.V. Co., Ltd., Almelo, the Netherlands) was used with a Cu target at a voltage of 40 KV, a current of 40 mA, a step width of 0.013°, and a scanning speed of 12 s/step in the 2θ scanning range from 4 to 30°.

#### 3.7.4. Fourier-Transform Infrared (FTIR) Spectra Analysis

For the FTIR analysis, 1 mg wheat bran fiber sample was mixed with 100 mg KBr and ground in an agate mortar. Then, the mixture was pressed into a flake, and the scanning was carried out in a Fourier-transform infrared spectrometer (Bruker Co., Ltd., Baden-Württemberg, Germany) with the scanning wavelength ranging from 4000 cm^−1^ to 400 cm^−1^ [51].

### 3.8. Dye Adsorption Properties of Fiber

For this analysis, 0.100 g of wheat bran fiber dye adsorbent was added into 100 mL of Congo red dye solution with the initial concentration of 100 mg/L at a certain temperature. After magnetically stirring for some time (5 min, 10 min, 20 min, 40 min, 60 min, 90 min, and 120 min), an appropriate amount of solution was collected and centrifuged. Subsequently, the absorbance of the supernatant was determined using a spectrophotometer (Shanghai Jinghua Technology Instrument Co., Shanghai, China) to calculate the dye concentration according to the standard curve. The absorption capacity was computed using the following equation:

(6) $$Q=\frac{(C_{0}-C_{t})\times V}{m}$$

where Q = absorption capacity (mg/g) of dye adsorbent; $C_{0}$ = the initial concentration (mg/L) of the solution; $C_{t}$ = the concentration (mg/L) of solution at time *t* (min); V = the volume (L) of the solution; m = the weight (g) of the adsorbent.

### 3.9. Adsorption Kinetics

The main steps of the adsorption kinetics revised from the reported approach [52] were as follows.

For this, 0.100 g modified XW 45 wheat bran fiber was added into 100 mL Congo red dye solution with the concentration of 100 mg/L at 30 °C, 40 °C, 50 °C, and 60 °C, respectively, and the blend was incubated in a water bath thermostatic oscillator (Changzhou Aohua Instrument Co., Ltd., Changzhou, China). Then, an appropriate amount of dye solution incubated for some time (10 min, 20 min, 30 min, 40 min, 60 min, 90 min, 120 min, and 180 min) was collected and centrifuged. After, the absorbance of the supernatant was determined to calculate the adsorption capacity and plot the adsorption kinetics curves at different temperatures.

The data from the adsorption kinetics study were, respectively, fitted to the pseudo-first-order and pseudo-second-order kinetics models.

The pseudo-first-order kinetic model is mainly applicable to the initial stage of adsorption, and its equation [53] is as follows:

(7) $$\ln\left(Q_{e}-Q_{t}\right)=lnQ_{e}-k_{1}t$$

where $Q_{e}$ = adsorption capacity (mg/g) of the adsorbent at equilibrium; $Q_{t}$ = adsorption capacity (mg/g) of the adsorbent at time t (min); $k_{1}$ = pseudo-first-order kinetic constant (min^−1^).

The pseudo-second-order kinetic model can be used to describe the entire adsorption process, and it is assumed that the entire adsorption process is mainly dominated by chemisorption [54]. Its equation [55] is as follows:

(8) $$\frac{t}{Q_{t}}=\frac{1}{k_{2}Q_{e}^{2}}+\frac{t}{Q_{e}}$$

where $Q_{e}$ = adsorption capacity (mg/g) of the adsorbent at equilibrium; $Q_{t}$ = adsorption capacity (mg/g) of the adsorbent at time t (min); $k_{2}$ = pseudo-second-order kinetic constant (g·mg^−1^·min^−1^).

### 3.10. Adsorption Isotherms

The research procedure of the adsorption isotherms revised from the published method [56] is as follows:

For the isotherms, 0.100 g modified XW 45 wheat bran fiber adsorbent was added into Congo red dye solution with concentrations of 50 mg/L, 100 mg/L, 150 mg/L, 200 mg/L, and 300 mg/L at 30 °C, 40 °C, 50 °C, and 60 °C, respectively. After the adsorption equilibrium, an appropriate amount of dye solution was collected and centrifuged. Then, the absorbance of the supernatant was determined so that the adsorption capacity could be calculated, and the curves at different temperatures could be drawn.

The data from the isothermal adsorption line were, respectively, fitted to the Langmuir and Freundlich isothermal adsorption models.

The Langmuir isothermal adsorption model assumes that the adsorbent is structurally homogenous, the energy of all adsorption sites is equivalent, and there is no interaction between the adsorbed substances. It applies to the monolayer adsorption process [57] and is usually used to describe the chemisorption process. The equation is as follows:

(9) $$\frac{C_{e}}{Q_{e}}=\frac{C_{e}}{Q_{m}}-\frac{1}{{K_{L}Q}_{m}}$$

where $Q_{e}$ = adsorption capacity (mg/g) of the adsorbent at equilibrium; $Q_{m}$ = maximum adsorption capacity (mg/g) of the adsorbent; $C_{e}$ = concentration of the dye solution at adsorption equilibrium (mg/L); $K_{L}$ = constant (L/mg) of the Langmuir model.

The Freundlich isothermal adsorption model is an empirical model, which is characterized by multilayer adsorption, and the energy of the active sites of the adsorbent is different [58]. Its equation is as follows:

(10) $$lgQ_{e}=lgK_{F}+\frac{1}{n}lgC_{e}$$

where $Q_{e}$ = adsorption capacity (mg/g) of the adsorbent at equilibrium; $C_{e}$ = concentration of the dye solution at the adsorption equilibrium (mg/L); $K_{F}$ = adsorption capacity constant ($L\cdot {mg}^{\frac{1}{n-1}}\cdot g^{-1}$) of the Freundlich model; n = Freundlich exponential constant.

### 3.11. Adsorption Thermodynamic Parameter

Based on the adsorption isotherm data, the thermodynamic parameters such as enthalpy change (∆H°), entropy change (∆S°), and Gibbs free energy change (∆G°) of the adsorption of Congo red dye by the modified XW 45 wheat bran fiber could be calculated. The calculation process mainly referred to the following equations [39]:

(11) $$K_{a}=\frac{Q_{e}}{C_{e}}$$

where $K_{a}$ = adsorption equilibrium constant; $Q_{e}$ = adsorption capacity (mg/g) of the adsorbent at equilibrium; $C_{e}$ = dye concentration at the adsorption equilibrium (mg/L).

(12) $$lnK_{a}=\frac{∆S{}^{\circ}}{R}-\frac{∆H{}^{\circ}}{RT}$$

where R = ideal gas constant (8.314 J·mol^−1^·K^−1^); ∆S° = entropy variation (J·mol^−1^·K^−1^); ∆H° = enthalpy variation (KJ/mol); T = temperature (K).

(13) ∆G°=∆H°−T∆S°

where ∆G° = the variation (KJ/mol) of Gibbs free energy. ∆S° and ∆H° were calculated from the fitted line after plotting T^−1^ by $lnK_{a}$. Then, ∆G° was calculated by substituting the values of ∆S° and ∆H° into Equation (13).

### 3.12. Statistical Analysis

All experiments were performed at least in triplicate, and their data were expressed as the mean ± standard deviation (SD). SPSS statistical 26 software was used for statistical analysis. The data were plotted using Origin 2018 software.

## 4. Conclusions

In order to promote the adsorption of anionic dyes and improve the utilization rate of wheat bran, wheat bran fibers from four different varieties were modified with NaOH, epichlorohydrin, and dimethylamine, which provided wheat bran fibers with larger pores and produced cationic modified absorbents. The maximum adsorption capacity of the unmodified wheat bran fiber for Congo red, an anionic azo dye, was only 20 mg/g within 120 min, while the maximum adsorption capacity of fiber could reach 93.46 mg/g within 120 min at room temperature. In the range of 30–60 °C, by heating, the adsorption of the modified wheat bran fiber could be accelerated, and the equilibrium adsorption capacity could be improved in the range of 50–300 mg/L. The study of adsorption kinetics and thermodynamics indicated that the dye was mainly adsorbed through chemisorption, and this process occurred spontaneously and endothermically. This study proved that the modification could improve the adsorption capacity of wheat bran fiber to dyes. It also provides a reference for the adsorption of dyes by agricultural by-products in an industrial environment, increases the added value of the wheat industry, and broadens the application of wheat bran.

## Figures and Tables

**Table 1:** Basic compositions and total dietary fiber contents of wheat bran.

Wheat Bran Source	Carbohydrates (g/100 g)	Protein (%)	Fat (g/100 g)	Ash (%)	Moisture (%)	Energy (KJ/100 g)	TDF (g/100 g)
FT	58.78 ± 0.06 ^c^	19.48 ± 0.08 ^a^	1.91 ± 0.02 ^a^	6.52 ± 0.03 ^a^	13.31 ± 0.02 ^d^	1401 ± 0.5 ^d^	58.49 ± 0.40 ^a^
XW 26	64.10 ± 0.07 ^a^	17.63 ± 0.03 ^b^	1.48 ± 0.03 ^b^	2.89 ± 0.06 ^c^	13.91 ± 0.03 ^b^	1444 ± 0.2 ^b^	29.67 ± 0.57 ^b^
XW 45	64.27 ± 0.33 ^a^	16.98 ± 0.19 ^c^	1.88 ± 0.01 ^a^	3.10 ± 0.01 ^b^	13.77 ± 0.12 ^c^	1451 ± 2.1 ^a^	27.77 ± 0.65 ^c^
KW 1701	62.99 ± 0.03 ^b^	17.02 ± 0.11 ^c^	1.88 ± 0.02 ^a^	3.13 ± 0.03 ^b^	14.99 ± 0.08 ^a^	1430 ± 1.8 ^c^	30.57 ± 0.61 ^b^

Values in the same column with different superscripts indicate significant differences (*p* < 0.05).

**Table 2:** Monosaccharide compositions of wheat bran fibers (Rha: rhamnose, Ara: arabinose, Gal: galactose, Glc: glucose, and Xyl: xylose).

Wheat Bran Source	Rha (%)	Ara (%)	Gal (%)	Glc (%)	Xyl (%)
FT	0.29	24.62	1.84	25.01	47.60
XW 26	0.00	10.94	0.96	64.67	23.42
XW 45	0.00	8.48	1.00	72.89	17.63
KW 1701	0.00	11.42	1.42	65.00	22.16

**Table 3:** Adsorption kinetic model and isotherm parameters of the modified XW 45 fiber for Congo red.

T (°C)	$Q_{e}$ (exp) (mg/g)	Pseudo-First-Order Model			Pseudo-Second-Order Model			Langmuir Isotherm			Freundlich Isotherm		
		$Q_{e}$ (calc) (mg/g)	$k_{1}$ (min^−1^)	R^2^	$Q_{e}$ (calc) (mg/g)	$k_{2}$ (g·mg^−1^ ·min^−1^)	R^2^	$Q_{m}$ (mg/g)	$K_{L}$ (L/mg)	R^2^	$K_{F}$	n	R^2^
30	88.70	99.27	0.0176	0.9959	158.73	4.547 × 10^−5^	0.9534	294.1	3.904 × 10^−2^	0.9263	31.19	2.262	0.6954
40	90.01	71.90	0.0212	0.9861	102.04	3.780 × 10^−4^	0.9978	344.8	1.165 × 10^−1^	0.9265	51.64	1.963	0.8182
50	95.74	70.60	0.0287	0.9669	106.38	5.471 × 10^−4^	0.9983	384.6	8.754 × 10^−2^	0.9028	41.21	1.639	0.8038
60	96.80	57.58	0.0323	0.9438	104.17	8.621 × 10^−4^	0.9985	370.4	1.343 × 10^−1^	0.8360	59.31	1.872	0.6183

**Table 4:** Thermodynamic parameters of the adsorption of Congo red by the modified XW 45 fiber.

T/K	∆H°/(KJ·mol^−1^)	∆S°/(J·mol^−1^·K^−1^)	∆H°/(KJ·mol^−1^)
303	55.10	198.61	−5.085
313			−7.071
323			−9.057
333			−11.04

## Data Availability

Data will be made available on request.
